# Boredom and Media Multitasking

**DOI:** 10.3389/fpsyg.2022.807667

**Published:** 2022-03-21

**Authors:** Allison C. Drody, Brandon C. W. Ralph, James Danckert, Daniel Smilek

**Affiliations:** Department of Psychology, University of Waterloo, Waterloo, ON, Canada

**Keywords:** state boredom, boredom proneness, media multitasking, attention, media use

## Abstract

Media multitasking entails simultaneously engaging in multiple tasks when at least one of the tasks involves media (e.g., online activities and streaming videos). Across two studies, we investigated one potential trigger of media multitasking, state boredom, and its relation to media multitasking. To this end, we manipulated participants’ levels of state boredom using video mood inductions prior to administering an attention-demanding 2-back task during which participants could media multitask by playing a task-irrelevant video. We also examined whether trait boredom proneness was associated media multitasking. We found no direct evidence that state boredom leads to media multitasking. However, trait boredom proneness correlated with greater amounts of media multitasking in Experiment 1, but not in Experiment 2. Surprisingly, in both experiments, post-task ratings of state boredom were equivalent across conditions, alerting us to the short-lived effects of video mood inductions and the boring nature of cognitive tasks.

## Introduction

The past decade has seen a dramatic increase in the use of multimedia devices such as smartphones and laptops. In fact, it is rare to find ourselves in the absence of these devices, as they are commonly used not only for work, but also for communication, information seeking, and entertainment (Hwang et al., 2014). However, the increased prevalence and possible uses of these devices raise concerns that they might also serve as distractions that impede our ability to focus on important tasks. In line with these concerns, a 2012 study found that college students spent, on average, 1 h per day using Facebook, 43 min per day searching the Internet, and 22 min per day checking emails *while completing schoolwork* (Junco and Cotten, 2012). Moreover, concerns surrounding the use of multimedia devices in the classroom has given rise to a number of studies aimed at controlling or reducing media use among students (Terry et al., 2016; Parry et al., 2020; Tassone et al., 2020). Beyond the classroom, media multitasking is likely a serious antecedent for distracted driving. One recent study found that 90% of respondents admitted to texting while driving at least some of the time (Hill et al., 2015). This tendency to simultaneously engage in multiple tasks, when at least one of the tasks involves media, has been referred to as media multitasking (e.g., Wang and Tchernev, 2012).

While media multitasking occurs frequently in daily life, there is substantial evidence to suggest that we are not effective media multitaskers. For instance, media multitasking has been shown to impair performance on perceptual and sustained attention tasks in the laboratory (e.g., Wang et al., 2012; Ralph et al., 2020, 2021). Media multitasking (e.g., by messaging or listening to podcasts) has also been shown to increase reading time (Fox et al., 2009; Bowman et al., 2010) and, in some cases, affect comprehension and memory of read material (Armstrong and Chung, 2000; Srivastava, 2013). Moreover, media multitasking in the classroom has been associated with poor learning of course content, especially when media is being used for course-unrelated activity (Hembrooke and Gay, 2003; Fried, 2008; Rosen et al., 2011; Wood et al., 2012; Demirbilek and Talan, 2018; Wammes et al., 2019; Jamet et al., 2020).

The fact that we are ineffective media multitaskers, but often choose to media multitask nonetheless, even in dangerous situations (e.g., Hill et al., 2015), highlights the importance of understanding the factors that motivate individuals to engage in this behavior. Some research has approached this issue by exploring the immediate needs that drive individuals to media multitask, often identifying desires to engage in routine activity, to seek enjoyment, to socialize, or to feel efficient by simultaneously engaging in multiple streams of information (Bardhi et al., 2010; Zhang and Zhang, 2012; Hwang et al., 2014; Kononova and Chiang, 2015; Lim and Shim, 2016; Kononova and Yuan, 2017; Robinson, 2017a,b; Lin, 2019; Su and Chen, 2020). People may also media multitask to feel a sense of control over their consumption of information or to satisfy cognitive needs related to learning and information seeking (Bardhi et al., 2010; Wang and Tchernev, 2012; Hwang et al., 2014; Kononova and Chiang, 2015; Robinson, 2017a, 2017b).

Other research has approached this issue by exploring the individual differences that increase one’s likelihood of media multitasking. Among the most common to emerge in this line of research have been sensation seeking, impulsivity and tendencies related to poor self-control (Jeong and Fishbein, 2007; Sanbonmatsu et al., 2013; Duff et al., 2014; Lim and Shim, 2016), including poor time management (Yang et al., 2015; Yang and Zhu, 2016) and difficulty regulating one’s use of media (Zhang and Rau, 2016). Many of these domains—most prominently, poor self-control, impulsivity, and sensation seeking—are known to be elevated in individuals prone to the experience of boredom (Kass and Vodanovich, 1990; Watt and Vodanovich, 1992; Dahlen et al., 2004; Struk et al., 2016; Isacescu et al., 2017). Despite this association, relatively little research has focused specifically on what the role of either state boredom or trait boredom proneness might be as an antecedent of media multitasking.

Boredom has been described as a negative state that arises when one’s desire to engage attention in an activity goes unfulfilled (Eastwood et al., 2012).^1^ Importantly, boredom has been theorized to signal rising opportunity costs associated with engaging in certain tasks at the expense of others, thereby motivating us to engage in more satisfying activity (Kurzban et al., 2013; Struk et al., 2020). Therefore, bored individuals with ready access to technology might engage in media multitasking to alleviate feelings of boredom. This seems particularly likely when one considers the variety of activities with which one could engage in using a single device. Consistent with this notion, students commonly cite boredom as a trigger of media multitasking (Rosen et al., 2013; Terry et al., 2016). Moreover, a study conducted by Ralph et al. (2020) demonstrated that participants were more likely to media multitask while completing a low-demand, boring task relative to a more challenging, high-demand task. They also found that boredom decreased when participants transitioned from a phase in which they were not allowed to media multitask while completing the task to one in which they were. Furthermore, this decrease in boredom was sharper among those completing the low-demand (boring) task compared to those completing the more challenging task. It is important to note here that Ralph et al. did not directly manipulate boredom but instead asked participants for retrospective evaluations of their boredom. Nevertheless, these results suggest that individuals may be more likely to media multitask under conditions that foster boredom as a means to alleviate the negative aspects of the state.

If in-the-moment feelings of boredom (i.e., state boredom) increase one’s likelihood of media multitasking, it is reasonable to expect that those high in trait boredom proneness, who are particularly prone to experiencing frequent and intense bouts of boredom (Tam et al., 2021), should media multitask more frequently than those who are less boredom prone. Indeed, those who report being bored during leisure time (leisure boredom; Iso-Ahola and Weissinger, 1990) have been shown to media multitask more frequently in daily life (Lin et al., 2020). Moreover, trait boredom proneness has been associated with mobile phone use while driving (Oxtoby et al., 2019), as well as media multitasking in daily life (Ralph et al., 2014).

One prominent limitation of the extant research that has explored the factors leading to media multitasking is that most have relied on participants’ retrospective reports of reasons for media multitasking (e.g., Rosen et al., 2013; Terry et al., 2016) or have linked trait measures to self-reported levels of media multitasking in daily life (e.g., Jeong and Fishbein, 2007; Sanbonmatsu et al., 2013; Ralph et al., 2014; Yang et al., 2015; Lim and Shim, 2016; Yang and Zhu, 2016; Zhang and Rau, 2016; Lin et al., 2020). No research that we are aware of has *experimentally* studied the antecedents of media multitasking. This may be due, in part, to the fact that many potential causes of media multitasking (e.g., low self-control or the need to socialize) are difficult to manipulate. However, the use of mood inductions could be instrumental in providing insight into the various affective states that lead to media multitasking. Video mood inductions in particular have been frequently used to study the influence of various affective states on cognition but have yet to be used in exploring the antecedents to media multitasking (e.g., Yeung et al., 2006; Smallwood et al., 2009; Martin and Kerns, 2011; Lerner et al., 2013; Scheibehenne and von Helversen, 2015).

Despite the abundance of evidence pointing to a relation between boredom and media multitasking, no research to date has experimentally investigated whether inducing boredom leads to media multitasking. To investigate whether state boredom leads to media multitasking, rather than relying on participants’ retrospective reports of reasons for media multitasking, we first manipulated participants’ levels of boredom by exposing them to a previously validated boredom or interest mood induction video (Merrifield and Danckert, 2014; Danckert and Merrifield, 2016). Following the mood induction, we administered a 2-back task (Kirchner, 1958) during which participants could media multitask. To assess the impact of the inductions on media multitasking, we employed a paradigm developed by Ralph et al. (2020), in which participants could turn a task-irrelevant video on or off at any point during the 2-back task by pressing a key on the keyboard. The number of trials during which the video was turned on served as our measure of media multitasking.

An additional goal of these experiments stemmed from the observation that, while trait boredom proneness has been associated with media multitasking in daily life (Ralph et al., 2014), no research has investigated the relation between trait boredom proneness and in-the-moment patterns of media multitasking. Therefore, in the present work, we also assessed whether trait boredom proneness correlated with media multitasking on the 2-back.

We hypothesized the following:

1. We predicted that participants who viewed the boring video would show higher levels of media multitasking during the 2-back relative to those who viewed the interesting video.
2. We anticipated that higher levels of trait boredom proneness would be associated with greater amounts of media multitasking on the 2-back.

## Experiment 1

### Method

#### Participants

Prior to commencing the study, we determined that we would halt data collection at the end of the semester. In all, 137 undergraduate students (32 male, 104 female, and 1 unknown) with an age range of 16–35 years old (*M_age_* = 19.49, SD = 2.25) took part in our study. Participants were recruited from a human participant pool at the University of Waterloo and participated in exchange for partial course credit. The study was approved by the University of Waterloo’s Office of Research Ethics and participants gave informed consent prior to participating.

#### Materials

##### Mood Inductions

To induce state boredom, participants watched a 4-min video of men hanging laundry and occasionally asking one another for a clothes peg. Interest was induced by presenting participants with a 4-min clip taken from the British Broadcasting Company’s (BBC) series *Planet Earth*, which portrayed colorful scenes of marine life accompanied by narration and music. These videos have been previously validated as effective inducers of boredom and interest, respectively (Merrifield and Danckert, 2014; Danckert and Merrifield, 2016).

##### State Boredom

State boredom was probed on three occasions during the experiment: Prior to watching the mood induction video, immediately after the mood induction, and following completion of the experimental task. Participants indicated their level of boredom by responding to the question, “How bored do you feel right now?” on a Likert scale ranging from 1 (not bored at all) to 7 (very bored).

##### Two-Back and Media Multitasking

Participants completed 468 trials (18 practice trials and 450 experimental trials) of a 2-back task. On each trial, a white letter (B, F, K, H, M, Q, R, X, or Z) appeared in the center of the screen for 500 ms against a black background. Each letter was followed by a white fixation cross that remained on the screen for 2000 ms. Participants were asked to press the spacebar when the letter present on the screen matched the letter presented two trials back. The 2-back contained a maximum of 78 target trials and 390 non-target trials. Practice trials were removed from our final analyses. Target frequency during the experimental trials occurred at a variable rate. There were 72 to 78 target trials per participant. Importantly, target frequency did not differ significantly between participants in the Boredom (*M* = 75.34, SD = 1.18) and Interest conditions (*M* = 75.10, SD = 1.22), *t*(127) = 1.13, *p* = 0.259. Performance on the 2-back was evaluated in terms of proportions of hits (i.e., proportions of spacebar presses in response to target trials) and false alarms (i.e., proportions of spacebar presses in response to non-target trials).

**Figure 1:**
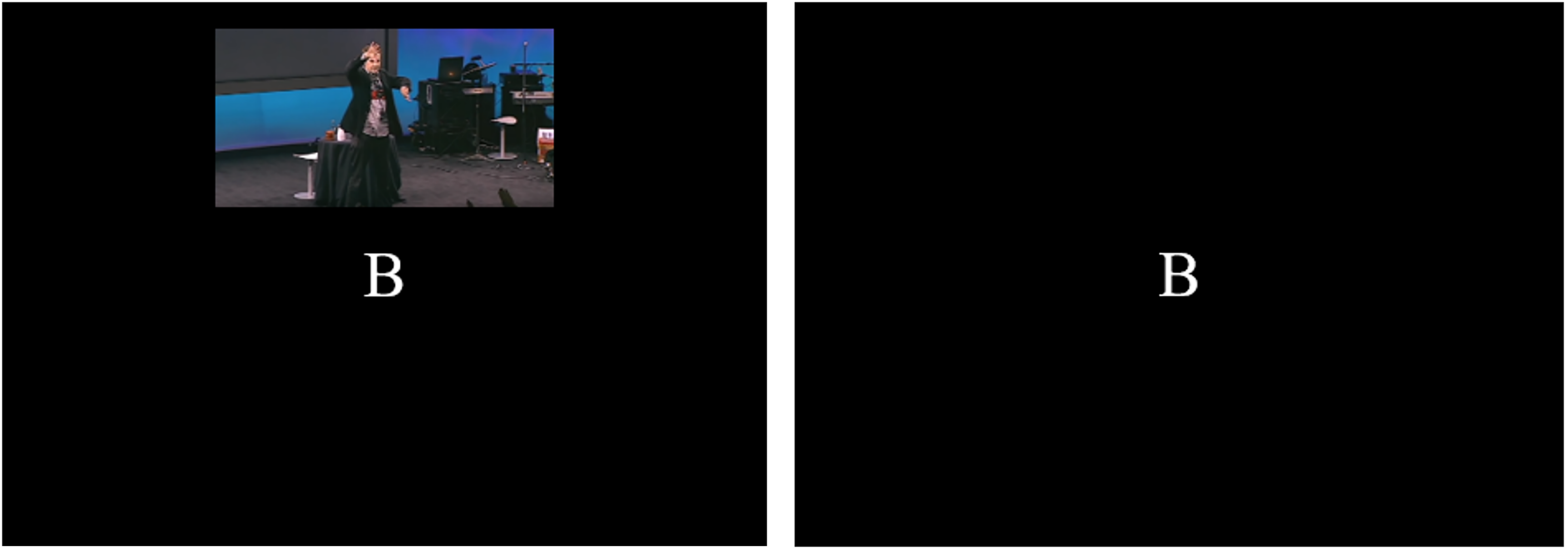
Depiction of the 2-back task when the video was being played (**left**) and when it was turned off (**right**).

Following the paradigm developed by Ralph et al. (2020), prior to commencing the 2-back, participants were informed that they could watch an optional video (a TED Talk by Keith Barry entitled “Brain Magic”) while completing the 2-back. The task began with the video turned off and participants could turn the video on or off at any time during the task by pressing the “t” key. If participants opted to play the video, it appeared in the upper, middle portion of the screen above the 2-back stimuli (Figure 1). If participants turned the video off and then on again, the video would resume from where participants left off. Participants received the following instructions regarding the optional video:

“While you complete this task, you will also have the opportunity to watch a video. There will be no test on the content of this video, and you are not required to watch it. However, you may watch the video while you do the 2-back, if you wish. The video will be turned off once you begin the task, but you may toggle the video on and off at your leisure, throughout the task, using the ‘t’ key (remember t for Toggle).”

The length of the video matched the length of the 2-back (approximately 20 min). The number of trials during which the video was being played served as our measure of media multitasking.

##### Post-Experiment Questions

After completing the experiment, participants were asked whether they had seen the mood induction videos before. Specifically, they were asked “Have you seen the video presented at the very beginning of this study before?” and could respond either “Yes, I have seen this video before” or “No, I have not seen this video before.” Additionally, to determine whether participants had seen the optional video in the past, we adapted the following question from a study by Ralph et al. (2021): “Have you seen the video presented during the experimental task (the 2-back) before?.” Participants could respond “Yes, I have seen this video before,” “No I have not seen this video before,” or “N/A, I did not watch the video at all.” Given the low percentage of participants who reported having previously seen the mood induction videos (10.85%) and the optional video (2.33%), these questions are not included in any further analyses.

##### Trait Boredom Proneness

Trait boredom proneness was assessed using the Short Boredom Proneness Scale (SBPS; Struk et al., 2017). The SBPS requires participants to rate their agreement with eight questions on a Likert scale ranging from 1 (strongly disagree) to 7 (strongly agree). Questions include, “I find it hard to entertain myself” and “Many things I have to do are repetitive and monotonous.” Scores on each item are summed, and may range from 8 to 56, with higher scores corresponding to higher levels of trait boredom proneness. Struk et al. (2017) report an internal consistency of 0.88.

#### Procedure

Participants were tested in groups of one to four, depending on the number enrolled for a given session. Each participant was seated at a desk and their view of other participants was obstructed by dividers placed between the desks. After participants provided informed consent, the experiment code was launched. All instructions for the experiment were provided on a computer screen and were accompanied by verbal instructions from a research assistant. Additionally, participants wore headphones throughout the experiment to reduce noise in the experiment room and to prevent them from hearing whether others were media multitasking by playing the video. At the start of the experiment, participants reported their level of boredom and were then randomly assigned to view the boredom (*n* = 72) or interest (*n* = 65) mood induction videos. Participants then provided post-induction ratings of boredom before completing the 2-back with the opportunity to media multitask. Following the 2-back, levels of boredom were probed once more, and participants were asked whether they had seen the mood induction and optional videos in the past. The entire experiment lasted approximately 25 min.

Trait boredom proneness scores were retrieved separate from the experimental session. The SBPS was included as part of a Mass Testing survey administered to the human participant pool at the University of Waterloo. SBPS scores were pulled from the Mass Testing survey after data collection was complete and linked to the current dataset.

### Results

#### Data Preprocessing

**Figure 2:**
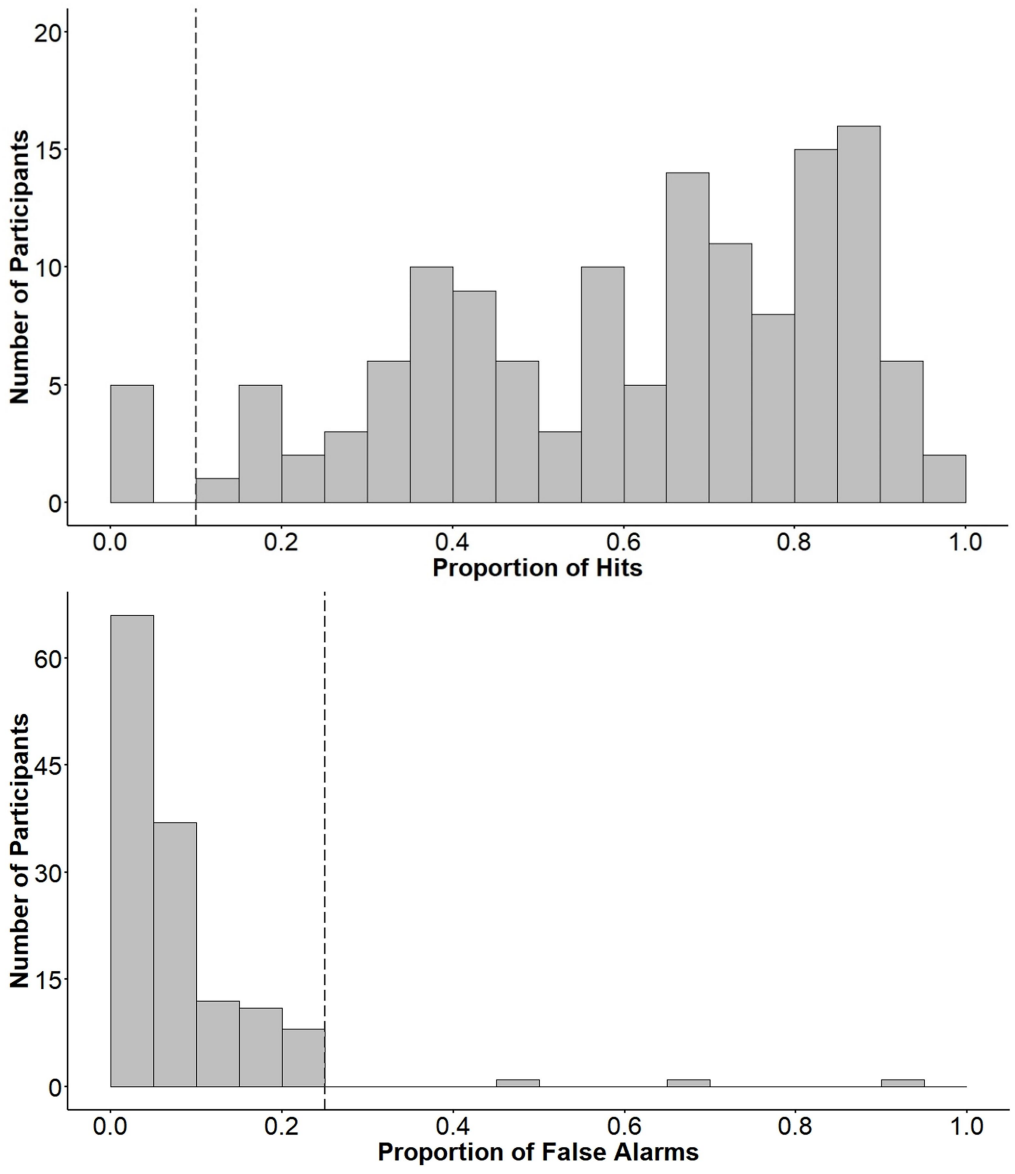
Histograms depicting participants’ proportions of hits (**top**) and false alarms (**bottom**). Vertical dashed lines mark the cut-off points for data removal.

Prior to acquiring the data for this experiment, we decided that we would visually inspect the distributions of participants’ proportions of hits and false alarms on the 2-back in order to remove participants with particularly poor performance that might be indicative of a failure to compete the task as instructed. Upon inspection of these distributions, we noted clear drop in each (Figure 2). Specifically, few participants scored less than 10% hits, except for a small subset of participants who scored either no hits or close to no hits on the 2-back. Additionally, very few participants scored more than 25% false alarms on the task. Therefore, participants with hit rates under 10% or false alarm rates over 25% were removed from our final dataset (Figure 2). Our final sample consisted of 129 participants (31 male and 98 female, *M_age_* = 19.50, *SD* = 2.30) with an age range of 16–35. There were 68 participants in the Boredom condition and 61 participants in the Interest condition. The full dataset, including outliers, can be found on the Open Science Framework (OSF) at the following link: https://osf.io/thna5/.

#### State Boredom

**Figure 3:**
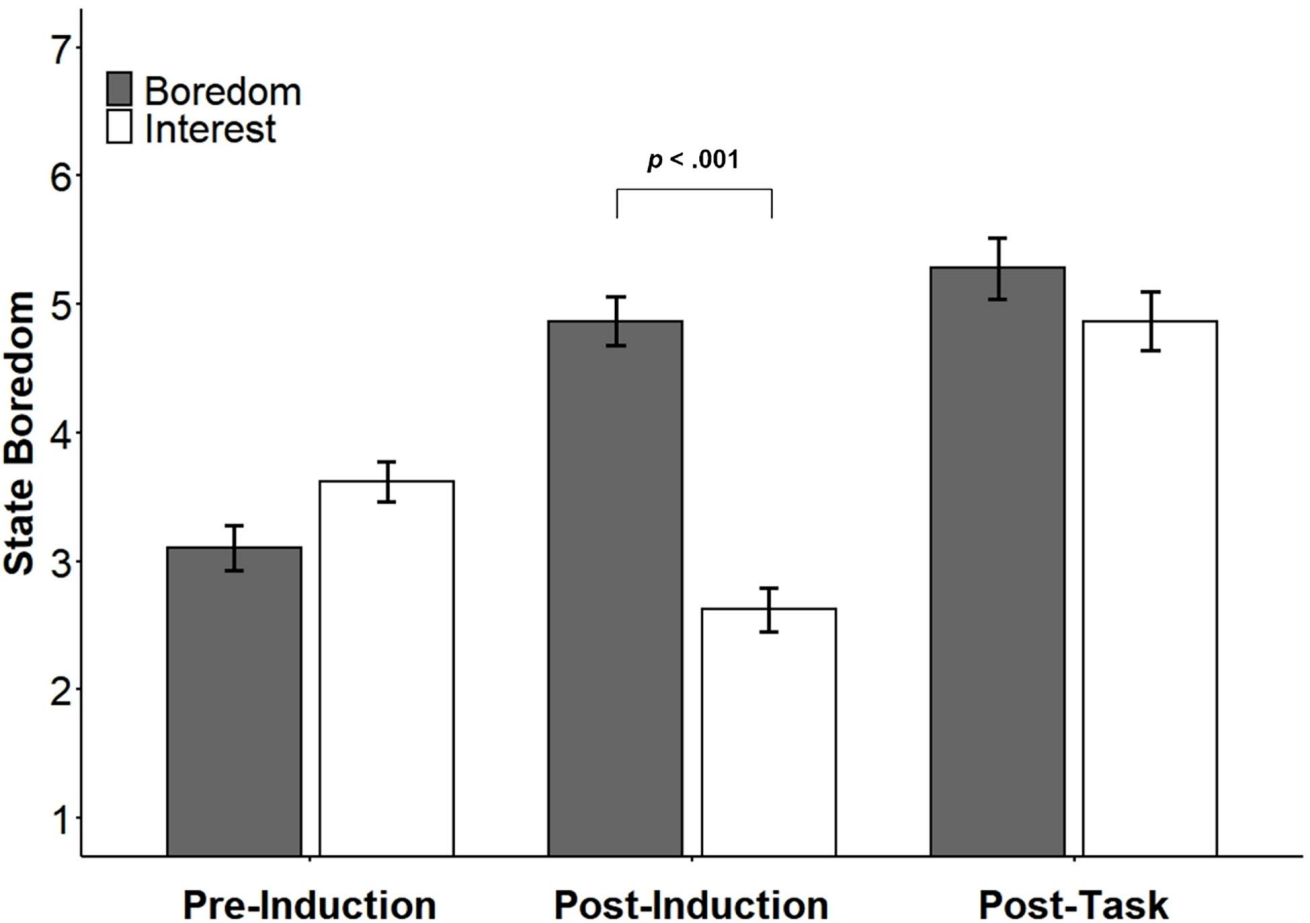
Mean (±SE) ratings of boredom before and after the mood inductions as well as following completion of the 2-back task among participants exposed to the boredom and interest mood inductions. Significant differences between conditions are indicated with brackets above the bars.

Mean boredom scores for each condition are shown in Figure 3. To examine changes in boredom over the course of the experimental session, ratings of state boredom were submitted to a 2 (Video: boredom or interest induction) × 3 (Time: pre-induction, post-induction, or post-task) mixed factorial ANOVA. Mauchly’s test indicated that the assumption of sphericity had been violated, *χ*^2^(2) = 14.56, *W* = 0.89, *p* = 0.001. Therefore, results are reported with degrees of freedom corrected using Greenhouse–Geisser estimates of sphericity (*ε* = 0.90). There was a main effect of condition, *F*(1, 127) = 15.20, *p* < 0.001, $\eta_{p}^{2}$ = 0.11, a main effect of time, *F*(1.80, 229.17) = 49.81, *p* < 0.001, $\eta_{p}^{2}$ = 0.28, and a significant interaction between condition and time, *F*(1.80, 229.17) = 30.55, *p* < 0.001, $\eta_{p}^{2}$ = 0.19.

Multiple comparisons adjusted using Tukey’s HSD confirmed that the mood inductions were successful at inducing their intended moods. Prior to the mood induction, there were no differences in ratings of boredom between those in the Boredom and Interest conditions (*p* = 0.416). Immediately following the mood induction, those who watched the boring video reported significantly higher boredom than they did before the induction (*p* < 0.001), while those who watched the interesting video reported significantly less boredom compared to their pre-induction levels of boredom (*p* = 0.002). Importantly, following the mood induction, those in the Boredom condition were significantly more bored than those in the Interest condition (*p* < 0.001). We also observed changes in boredom from the start to the end of the 2-back task. Participants in the Interest condition experienced a significant increase in boredom following completion of the task (*p* < 0.001), whereas those in the Boredom condition did not (*p* = 0.557). Post-task ratings of boredom did not differ between groups (*p* = 0.675).

#### Media Multitasking

**Figure 4:**
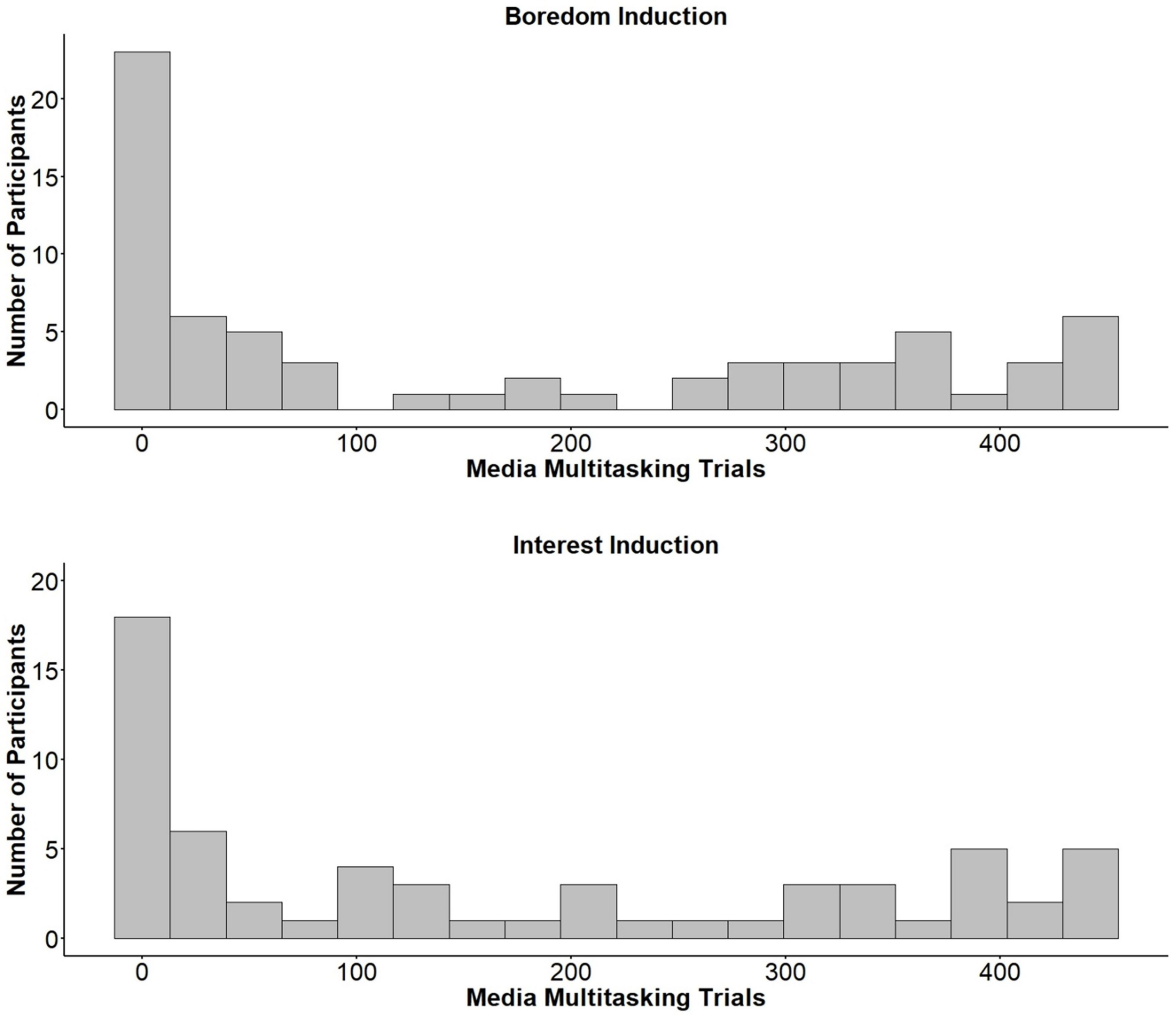
Histograms showing the total number of participants who media multitasked for a given number of trials. Histograms are split based on whether participants were exposed to the boredom (**top**) or interest (**bottom**) induction video.

Due to the highly skewed distribution of the media multitasking data (Figure 4), a Wilcoxon rank-sum test was used to compare rates of media multitasking between groups. Results revealed that rates of media multitasking did not differ significantly between those who had undergone the boredom (*Mdn* = 65) and interest (*Mdn* = 117) inductions, *W* = 1935.5, *p* = 0.512, *r* = −0.28. Additionally, no difference was found between the Boredom (*Mdn* = 44) and Interest (*Mdn* = 66) conditions when comparing on which trial participants first played the video, *W* = 1007, *p* = 0.226, *r* = −0.12. Finally, we found no difference between conditions (*Mdns* = 2) in terms of the number of times participants turned the video on or off during the 2-back, *W =* 2002, *p* = 0.731, *r* = −0.03.

#### Trait Boredom Proneness and Media Multitasking

**Figure 5:**
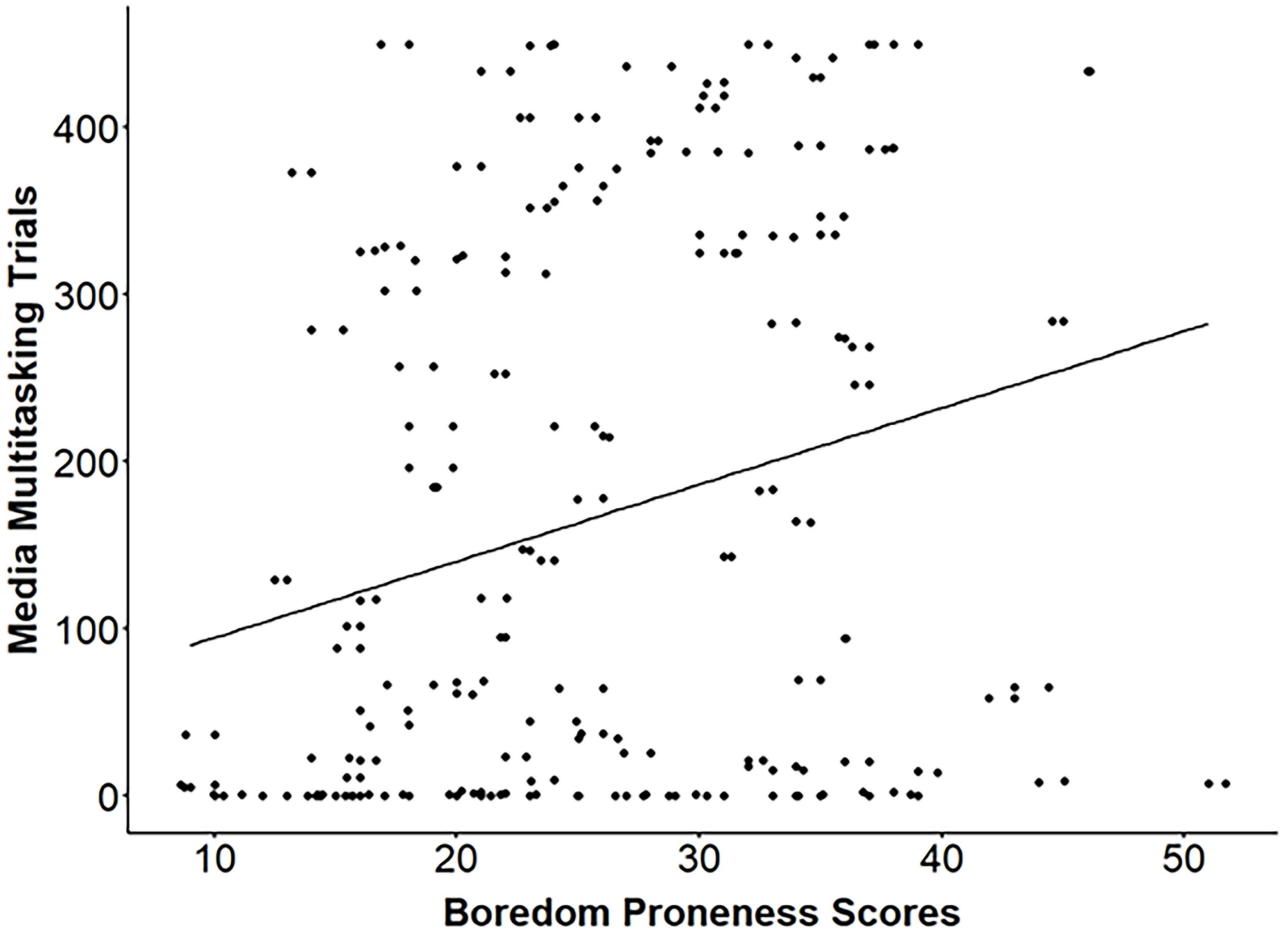
Scatterplot illustrating the relationship between trait boredom proneness and the number of trials spent media multitasking.

Given the skewed nature of the media multitasking data (Figure 4), a Spearman rank-order correlation was performed to examine whether trait boredom proneness was associated with higher rates of media multitasking on the 2-back. SBPS scores could not be traced for one participant and were therefore not included in the present analysis. The SBPS demonstrated good internal consistency (*α* = 0.89). We found a significant correlation between trait boredom proneness and the number of 2-back trials during which participants played the task-irrelevant video, *r_s_*(126) = 0.28, *p* = 0.001 (Figure 5). Trait boredom proneness did not relate, however, to the time that participants first played the video, *r_s_*(126) = −0.10, *p* = 0.309, or the number of times participants turned the video on or off, *r_s_*(126) = 0.13, *p* = 0.138.

#### 2-Back Performance

Descriptive statistics of performance on the 2-back are shown in Table 1. Levene’s test indicated that variances were unequal between conditions, *F* = 4.99, *p* = 0.027. Therefore, a Welch’s *t*-test was conducted to assess whether proportion of hits differed significantly by condition. Results revealed that proportions of hits on the 2-back did not differ significantly between those in the Boredom or Interest conditions, *t*(117.15) = 0.28, *p* = 0.779, *d* = 0.05. Due to the skewed distribution of proportion of false alarms (Table 1), rates of false alarms were compared using a Wilcoxon rank-sum test. Proportion of false alarms did not differ between conditions, *W* = 2444.5, *p* = 0.081, *r* = 0.15.

**Table 1:** Proportions of hits and false alarms as a function of mood induction condition.

	Mood Induction	Mean (SD)	Median	Skew	Kurtosis
Proportion of hits	Boredom	0.63(0.20)	0.68	−0.41	−0.72
	Interest	0.61(0.24)	0.66	−0.42	−1.21
Proportion of false alarms	Boredom	0.08(0.06)	0.06	1.18	0.55
	Interest	0.07(0.06)	0.04	1.40	1.13

### Discussion

In our first experiment, we investigated whether state boredom and trait boredom proneness led to media multitasking during an attention-demanding 2-back task. While our mood inductions were successful at inducing their intended moods, manipulating participants’ levels of boredom had no effect on subsequent rates of media multitasking. However, consistent with our second hypothesis, we found that trait boredom proneness correlated with greater amounts of media multitasking.

An unexpected outcome of this experiment was that, although our mood inductions were initially effective, post-task ratings of boredom were equivalent between participants who had undergone either mood induction. This finding raises the possibility that the effects of our mood inductions were simply too short-lived to lead to any significant differences in media multitasking between groups. One factor that might have shortened the duration of the effects of our mood inductions could have been the boring nature of the 2-back task itself. Recall that those in the interest condition experienced a significant increase in boredom from the start to the end of the 2-back, bringing their ratings to the same levels as those in the boredom mood induction group (Figure 3). Perhaps the convergence of post-task ratings of boredom occurred because the 2-back task itself was sufficiently boring to rapidly overpower any effect of the interest mood induction. These findings motivated us to explore whether manipulating boredom might have a short-term effect on media multitasking.

## Experiment 2

In our second experiment, we employed a similar paradigm to the one used in Experiment 1 to further investigate whether state boredom leads to media multitasking. To account for the possibility that our inability to detect an effect of our boredom manipulation in Experiment 1 was hindered by the short-lived effects of our mood inductions and to maximize our chances of detecting an effect in the present study, the length of the 2-back task was shortened to only 108 trials (i.e., approximately one quarter of the length of the task used in Experiment 1). We predicted that inducing state boredom would lead to short-term increases in media multitasking. We also aimed to replicate the associations found in Experiment 1 between trait boredom proneness and media multitasking.

### Method

#### Participants

Based on the samples acquired in past work on volitional media multitasking (Ralph et al., 2021), prior to commencing data collection, we determined that we would collect a total of 160 participants. Thus, 160 participants (80 per condition) were recruited through Amazon Mechanical Turk and participated in exchange for $2.00 paid to their Mechanical Turk account. In order to take part in our study, participants were required to have a hit rate of at least 97% and a minimum approval rate of 1,000.^2^ The study was approved by the University of Waterloo’s Office of Research Ethics and participants gave informed consent prior to participating.

#### Post-task Compliance Check

To address concerns that participants might not be fully attending to our online experiment, we included a post-task compliance check which asked participants whether they had engaged in activities unrelated to the experiment while taking part in our study. Participants received the following question immediately following completion of the 2-back:

“While completing this study, were you engaged in any media-related activities outside of the contents of the experiment (e.g., attending to content in another browser, listening to music or using a smartphone/tablet while completing the study)?

Yes.

No, I did not engage in any activities outside of the contents of this study.

No, but I was engaged in other, media-unrelated activities while completing this study.”

Those who responded that they had engaged in activities unrelated to our experiment during the session were removed from our final dataset.

#### Materials and Procedure

The materials and procedure in this experiment were nearly identical those in Experiment 1, with some exceptions. After viewing the boredom or interest induction video, participants were given the option to media multitask while completing a 2-back task that lasted only 108 trials (18 practice trials and 90 experimental trials), or 4.5 min. Target frequency during the experimental trials occurred at a variable rate, with a range of 12–18 target trials per participant. Importantly, target frequency did not differ significantly between those in the Boredom (*M* = 15.46, SD = 1.19) and Interest conditions (*M* = 15.36, SD = 0.95), *t*(125) = 0.53, *p* = 0.598. After participants completed the 2-back, they responded to the post-task compliance check in addition to two questions asking whether they had previously seen the mood induction and optional videos. As in Experiment 1, few participants reported having seen the mood induction videos (7.09%) and the optional video (1.57%) in the past. Therefore, these questions will not be included in any further analyses. The entire experiment lasted approximately 7 min.

### Results

#### Data Preprocessing

A total of 26 participants, who reported having engaged in external tasks while completing the study, as measured by our post-task compliance check, were removed prior to analysis of the data. Additionally, we applied the same data removal criteria used in Experiment 1 to remove participants with particularly poor performance on the 2-back. Therefore, participants with hit rates under 10% and false alarms over 25% were removed from our analyses. Our final sample consisted of 127 participants. There were 63 participants in the Boredom condition and 64 in the Interest condition. The full dataset for this experiment is available on OSF.^3^

#### State Boredom

**Figure 6:**
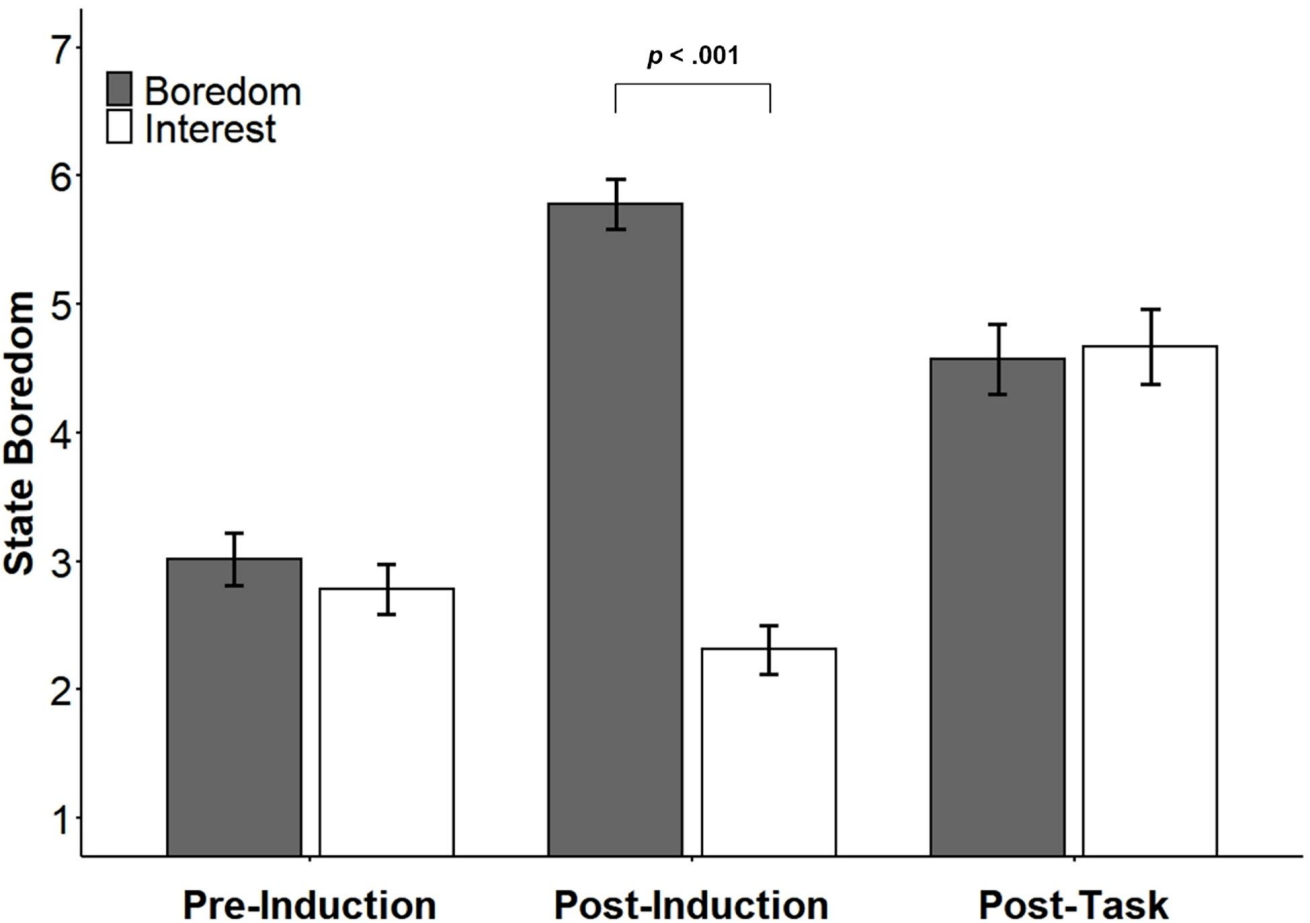
Mean (±SE) ratings of state boredom for those who viewed the boredom or interest induction video. Ratings of boredom were taken before and after the mood induction as well as following completion of the 2-back task. Significant differences between conditions are indicated with brackets above the bars.

Mean ratings of state boredom for those in the Boredom and Interest conditions are illustrated in Figure 6. To assess changes in boredom throughout the experimental session, ratings of state boredom were submitted to a 2 (Video: boredom or interest induction) x 3 (Time: pre-induction, post-induction, or post-task) mixed factorial ANOVA. Mauchly’s test indicated that the assumption of sphericity had been violated, *χ^2^*(2) = 10.72, *p* = 0.005. Therefore, degrees of freedom were adjusted using Greenhouse–Geisser estimates of sphericity (*ε* = 0.92). There was a main effect of Video, *F*(1, 125) = 21.44, *p* < 0.001, $\eta_{p}^{2}$ = 0.15, a main effect of Time, *F*(1.85, 231.02) = 54.92, *p* < 0.001, $\eta_{p}^{2}$ = 0.31, and a significant interaction between Video and Time, *F*(1.85, 231.02) = 69.20, *p* < 0.001, $\eta_{p}^{2}$ = 0.36.

Multiple comparisons adjusted using Tukey’s HSD confirmed that our mood inductions were successful. Prior to the mood induction, boredom levels were comparable between those in the Boredom and Interest conditions (*p* = 0.979). Following the mood induction, participants in the Boredom condition experienced a significant increase in boredom (*p* < 0.001), whereas those in the Interest condition did not experience any change in their levels of boredom (*p* = 0.352). Importantly, immediately after the mood induction, those who viewed the boring video were significantly more bored than those who viewed the interesting video (*p* < 0.001). Relative to pre-task ratings of boredom, those in the Interest condition became significantly more bored following completion of the 2-back while those in the Boredom condition became less bored (*ps* < 0.001). Post-task ratings of boredom were equivalent between conditions (*p* = 0.9996).

#### Media Multitasking

**Figure 7:**
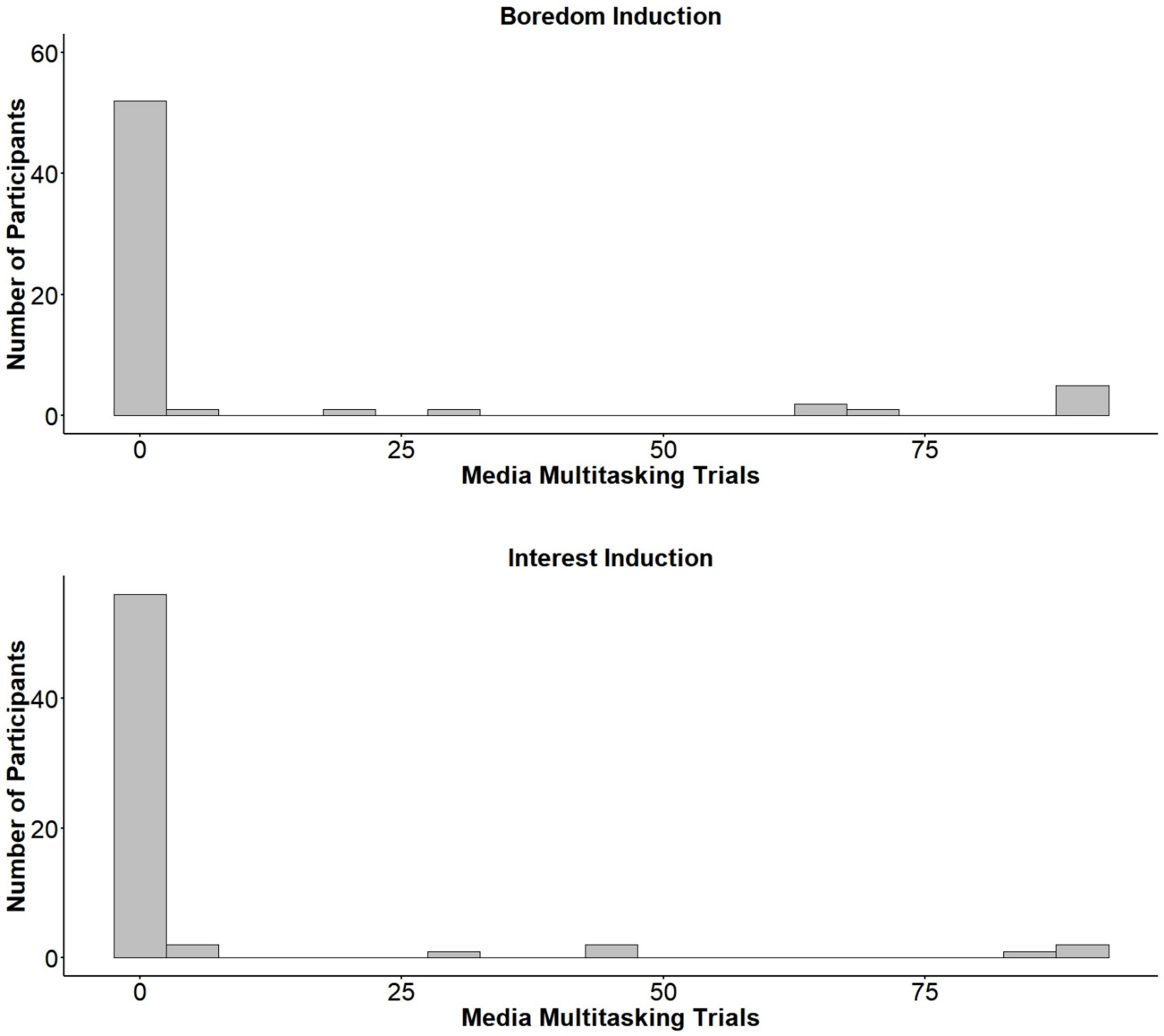
Histograms showing the total number of participants who media multitasked for a given number of trials, based on whether they viewed the boredom (**top**) or interest (**bottom**) induction videos.

Given the skewed nature of the media multitasking data (Figure 7), a Wilcoxon rank-sum test was used to assess whether media multitasking differed based on mood induction condition. There was no significant difference in rates of media multitasking between conditions (*Mdns* = 0), *W* = 2106.5, *p* = 0.525, *r* = 0.06. Further, there were no differences between groups when comparing on which trial participants first turned on the video (*Mdns* = 2), *W* = 75.5, *p* = 0.835, *r* = 0.05, or the number of times participants switched the video on or off (*Mdns* = 0), *W* = 200.5, *p* = 0.905, *r* = −0.01.

#### Trait Boredom Proneness and Media Multitasking

As in Experiment 1, we investigated whether trait boredom proneness correlated with media multitasking during the 2-back. We note that the results of the following correlations should be interpreted with caution, as there were very few instances of media multitasking during the 2-back (Figure 7). Data for three participants who did not respond to all items on the SBPS are not included in our analyses. The SBPS showed good internal consistency (*α* = 0.90). Results of several Spearman rank-order correlations revealed that trait boredom proneness did not correlate with media multitasking during the 2-back, *r_s_*(122) = 0.03, *p* = 0.728, the trial on which participants first played the video, *r_s_*(122) = 0.04, *p* = 0.849, or the number of times participants turned the video on or off *r_s_*(122) = 0.02, *p* = 0.864.

#### 2-Back Performance

Proportions of hits and false alarms for those in the Boredom and Interest conditions can be found in Table 2. Due to the skewed distribution of proportion of hits (Table 2), scores for this variable were submitted to a Wilcoxon rank-sum test. Proportion of hits did not differ significantly between groups, *W* = 2,139, *p* = 0.554. Proportions of false alarms were also compared between groups. Levene’s test indicated that variances were unequal between groups, *F*(1, 125) = 4.78, *p* = 0.031. Therefore, a Welch’s *t*-test was conducted to assess whether proportion of false alarms differed by condition. There was no difference in proportions of false alarms between conditions, *t*(122.39) = −0.47, *p* = 0.639, *d* = 0.08.

**Table 2:** Proportions of hits and false alarms as a function of mood induction condition.

	Mood Induction	Mean (SD)	Median	Skew	Kurtosis
Proportion of hits	Boredom	0.78(0.19)	0.80	−1.50	2.71
	Interest	0.74(0.24)	0.80	−1.17	0.54
Proportion of false alarms	Boredom	0.08(0.06)	0.07	0.65	−0.06
	Interest	0.08(0.07)	0.06	0.60	−0.87

## Discussion

In Experiment 2, we sought to investigate whether inducing state boredom would lead to short-term increases in media multitasking. We were also interested in whether trait boredom proneness would correlate with media multitasking on the 2-back, as was the case in Experiment 1. Rates of media multitasking during the 2-back were extremely low, and we found no significant difference in media multitasking between those who underwent the boredom and interest inductions. Moreover, we failed to replicate the correlation found in Experiment 1 between trait boredom proneness and media multitasking on the 2-back. As in Experiment 1, post-task ratings of state boredom increased from pre-task levels among those in the Interest condition despite our relatively short (~4.5 min) task. Furthermore, post-task ratings of boredom were equivalent between groups. Taken together, these findings suggest that the effects of our mood inductions might have been too short-lived to lead to any differences in media multitasking between groups.

## General Discussion

Across two experiments, we explored whether inducing state boredom would lead to media multitasking. We also examined whether individual differences in trait boredom proneness were associated with greater amounts of media multitasking during attention-demanding tasks. In both studies, manipulating participants’ levels of state boredom did not lead to differences in rates of media multitasking between groups. Therefore, we found no direct evidence to support the notion that state boredom leads to media multitasking in contrast to prior research suggesting that individuals media multitask out of boredom (e.g., Rosen et al., 2013; Terry et al., 2016; Ralph et al., 2020). Regarding trait boredom proneness, we found a positive relation between scores on the SBPS and media multitasking in Experiment 1. We found no significant relation between these variables, however, in Experiment 2.

Previous research has shown that boredom-prone individuals often have difficulty regulating their media use (e.g., Skues et al., 2016; Elhai et al., 2018; Wegmann et al., 2018) and media multitask frequently in daily life (Ralph et al., 2014). The relation between trait boredom proneness and media multitasking found in Experiment 1 but not Experiment 2 provides further, albeit spotty, evidence for a link between trait boredom proneness and media multitasking by demonstrating a relation between individual differences in boredom proneness and in-the-moment patterns of media multitasking. We believe that our failure to find an association between trait boredom proneness and media multitasking in Experiment 2 might stem from the fact that there were few instances of media multitasking in Experiment 2 (the median number of media multitasking trials was 0).

Low rates of media multitasking in our second experiment may be explained by the samples collected in each study. Whereas our sample in Experiment 1 consisted of undergraduate students participating in exchange for course credit, participants in Experiment 2 were recruited through Amazon Mechanical Turk and took part in our study in exchange for monetary reward. This latter sample might differ in meaningful ways from the university sample. For example, given that Mechanical Turk participants represent a group that voluntarily takes part in studies that may be considered monotonous in exchange for monetary reward, these individuals may be less susceptible to boredom while completing such tasks, and thus less likely to engage in behaviors such as media multitasking during online studies. Consistent with this explanation, participants in Experiment 2 generally scored lower on our measure of trait boredom proneness (*M* = 21.72, SD = 10.97) than participants in Experiment 1 (*M* = 25.50, SD = 8.90; *t*(250) = 2.99, *p* = 0.003). Further evidence in support of this explanation comes from studies suggesting that high-reputation Mechanical Turk workers (i.e., those with approval ratings above 95%, similar to our sample in Experiment 2) remain attentive during online studies (Peer et al., 2014) and might be even more attentive than participants recruited through university participant pools (Hauser and Schwarz, 2016). Therefore, it may be that differences in our findings across studies stem from differences in trait boredom proneness between our two samples. Another possibility for why rates of media multitasking were low in our second experiment is that our online sample in Experiment 2 did not complete the study in a laboratory setting. As a result, participants in Experiment 2 could have been exposed to a variety of distractors (e.g., people in their environment and background noise) not typically present in the laboratory. Exposure to these distractors might have reduced participants’ desire to engage with the video while completing the 2-back. Finally, low rates of media multitasking in Experiment 2 might simply be explained by our shortened 2-back task. Perhaps individuals did not feel inclined to turn on the video because the 2-back was only expected to last 5 min.

One limitation of our experiments is that we did not ask participants about their thoughts or experiences relating to the task-irrelevant video. Thus, it is unclear whether factors such as participants’ curiosity about, or interest in, the video influenced their media multitasking behaviors. Additionally, we did not measure any traits that might affect one’s willingness to play the video. Openness to experience, for example, might make one especially likely to turn the video on, regardless of which mood induction they viewed. Consistent with this notion, Jach and Smillie (2021) found that individuals high in trait curiosity, a facet of openness to experience (Silvia and Christensen, 2020), were more likely to seek task-irrelevant information than participants who were low in this trait. Therefore, future studies could explore the relation between boredom and media multitasking using a paradigm similar to the one employed in the present experiments, while controlling for factors such as curiosity or openness to experience. That rates of media multitasking did not vary based on whether participants underwent a boredom or interest induction is surprising given that prior research has suggested that boredom motivates media multitasking (e.g., Rosen et al., 2013; Terry et al., 2016). Nevertheless, our findings are intriguing, as they raise the possibility that the effects of our video mood inductions were too short-lived to lead to significant group differences in media multitasking. Indeed, while our videos were initially successful at inducing their intended moods in both experiments, ratings of post-task boredom increased relative to pre-task levels among those in the Interest condition such that post-task ratings of boredom were equivalent across groups. Thus, it appears that the effect of the interest induction did not persist for the duration of the 2-back. This finding was particularly striking in Experiment 2, in which the 2-back task was shortened to last only 108 trials.

Our results are congruous with a small number of studies demonstrating that the effects of mood inductions tend to be short-lived (e.g., Frost and Green, 1982; Isen and Gorgoglione, 1983; Hunter and Eastwood, 2018). Importantly, we cannot speak to the duration of our boredom induction, as our mood induction videos were followed by a task that appeared to function well as a boredom inducer in its own right. An alternative approach to studying the influence of boredom on media multitasking may be to offer participants the opportunity to media multitask while watching the mood induction videos and not after having watched them. However, a shortcoming of this study would be that the primary task would no longer be held constant between conditions. Therefore, one risks confounding the nature of the video with the experience of boredom. Another approach might be to simply replace the 2-back task with a more interesting task. However, this would not solve the problem either, as it may be likely to negate the effects of our boredom induction. Therefore, not only is it possible that the effects of mood inductions are short-lived, but their effects might also be overpowered by moods invoked by other tasks.

Related to this notion, our findings regarding increases in boredom in the Interest condition and comparable post-task ratings of boredom between groups also highlight the potential for laboratory tasks to rapidly induce boredom. Interestingly, Hunter and Eastwood (2018) found results similar to our own. Specifically, their manipulation of boredom, which involved the use of video mood inductions different from the ones used in the present experiments, was initially effective in varying participants’ levels of boredom; however, levels of boredom were equivalent between groups following only 243 trials of a sustained attention task. While it is no surprise that many cognitive tasks employed in psychological research tend to be monotonous and lead to feelings of boredom (e.g., Scerbo, 1998; Hunter and Eastwood, 2018), these results suggest that task-induced boredom may be a confound in studies in which it is unaccounted for. Therefore, when employing cognitive tasks to observe the consequence of mood inductions, one should consider the duration of the tasks, as well as their capacity to induce boredom. Furthermore, it would be useful to probe induced moods throughout an experiment rather than sampling levels of targeted moods only before and after the induction to ensure that the inductions are exerting their intended effects.

To conclude, while manipulating participants’ levels of boredom did not influence their levels of media multitasking in the present studies, the use of mood inductions in our experiments emphasizes the importance of being cautious when employing video mood inductions, the effects of which may be extremely short-lived. Moreover, our findings suggest that experimental tasks are strong inducers of boredom and should warn researchers of the potential dangers of employing such tasks when boredom is not considered.

## Data Availability Statement

The datasets presented in this study can be found in online repositories. The names of the repository/repositories and accession number(s) can be found at: The Open Science Framework at https://osf.io/thna5/.

## Ethics Statement

The studies involving human participants were reviewed and approved by the Office of Research Ethics at University of Waterloo. The patients/participants provided their written informed consent to participate in this study.

## Author Contributions

The first draft of the manuscript was written and information presented in this manuscript is based on data collected for a master’s thesis completed by AD. Material preparation and collection and analysis of the data were performed by AD and BR. The experiments were programmed by BR. All authors contributed to the article and approved the submitted version.

## Funding

This research was supported by the Discovery Grant from the Natural Sciences and Engineering Research Council of Canada awarded to JD (no. 50503-10762) and DS (no. 2019-04071), as well as the Canadian Graduate Scholarship Master’s from the Social Sciences and Humanities Research Council awarded to AD.

## Conflict of Interest

The authors declare that the research was conducted in the absence of any commercial or financial relationships that could be construed as a potential conflict of interest.

## Publisher’s Note

All claims expressed in this article are solely those of the authors and do not necessarily represent those of their affiliated organizations, or those of the publisher, the editors and the reviewers. Any product that may be evaluated in this article, or claim that may be made by its manufacturer, is not guaranteed or endorsed by the publisher.

## Footnotes

^1^We acknowledge that there are other potential accounts of boredom, including opportunity costs (Danckert et al., 2018), prompts for exploratory action (Bench and Lench, 2013; Elpidorou, 2014), and responses to a lack of meaning (Westgate and Wilson, 2018). In this paper, we focus on the attentional account of boredom (Eastwood et al., 2012); however, we note that the results of the studies presented here are pertinent to whichever model of state boredom one considers.

^2^This experiment did not include bot checks; however, our recruitment criteria were intended to reduce our likelihood of including bots and non-compliant participants. Moreover, because our experimental task asked participants to press the spacebar in response to target trials and we would expect bots to respond to all or none of the trials, it is likely that bots would have been removed in data preprocessing.

^3^ https://osf.io/thna5/
